# Environmental and Parental Influences on Offspring Health and Growth in Great Tits (*Parus major*)

**DOI:** 10.1371/journal.pone.0069695

**Published:** 2013-07-30

**Authors:** Simon R. A. Pickett, Sam B. Weber, Kevin J. McGraw, Ken J. Norris, Matthew R. Evans

**Affiliations:** 1 School of Biosciences, University of Exeter, Penryn, Cornwall, United Kingdom; 2 School of Life Sciences, Arizona State University, Tempe, Arizona, United States of America; 3 School of Agriculture, Policy and Development, The University of Reading, Reading, Berkshire, United Kingdom; Hungarian Academy of Sciences, Hungary

## Abstract

Sexual selection requires both that there is heritable variation in traits related to fitness, and that either some of this variation is linked to traits of the parents, and/or that there are direct benefits of choosing particular individuals as mates. This suggests that if direct benefits are important offspring performance should be predicted by traits of the rearing adults. But if indirect benefits are more significant offspring performance should be predicted by traits of the adults at the nest-of-origin. We conducted cross-fostering experiments in great tits (*Parus major*) over four years, in two of which we manipulated environmental conditions by providing supplemental food. In a third year, some nestlings were directly supplemented with carotenoids. Nestlings in broods whose rearing adults received supplemental food were heavier and had improved immune responses even when controlling for body mass. Nestling immune function was related to measures of the yellow plumage color of both the rearing male and the putative father. Nestling body mass was influenced by the coloration of both the rearing female and the genetic mother. Our results suggest that features of both their social and putative genetic parents influence nestling health and growth. From this it would appear that females could be gaining both direct and indirect benefits through mate choice of male plumage traits and that it would be possible for males to similarly gain through mate choice of female traits.

## Introduction

In the natural setting, genetic effects, maternal effects, the effect of parental effort and environmental effects will often be confounded, making it difficult to discern their individual influences, for a discussion of this issue see Kruuk & Hadfield [1]. This is because adult animals will vary in their parenting ability and/or in their willingness to invest in the current breeding attempt and these are likely to covary with their individual quality, so we might expect adults of high genetic quality to be better parents [2] and perhaps also to occupy high-quality breeding sites [3]. However to test certain hypotheses we need to determine the extent of the influence of genetic and environmental factors on offspring. Sexual selection theory assumes that there is a link between the degree of expression of a trait used in mate choice and the potential benefits to the choosy sex of selecting a partner displaying that trait [4]. It has been understood for a long time that such benefits can be direct (e.g. improved parenting ability, access to resources, etc.) and/or indirect (good genes) [4]. If either sex gains direct benefits by choosing a high-quality mate, one would expect that ornamental traits in the chosen sex should reflect the quality of the environment in which offspring are reared. In contrast, if indirect benefits are gained, one would expect to see ornaments reflecting their genetic quality.

Heritability measured in laboratory conditions does not always provide good estimates of the contribution of genetic sources of variation in field situations [5], because environmental variation magnifies phenotypic variance in the field [6]. Altricial animals offer a unique opportunity to disentangle the effects of parents and environment on offspring performance in the field, as newly hatched offspring can be transferred between broods in a manner that would be almost inconceivable for many other organisms with similar levels of parental care. However, it remains impossible to break the link between early maternal effects (i.e. those that act before the offspring are moved between broods) and genetic effects. Despite this caveat moving offspring at an early stage of development gets close to achieving the goal of disassociation of genetic and environmental effects [7]. In such an experiment one would wish to assess a good proxy measure of fitness in the offspring as the dependent variable [8]–[10], the majority of studies have focussed on body mass while a few have also assessed immune function both of which are relatively easy to measure in nestling birds.

Significant nest-of-origin (genetic or early maternal) effects on nestling immune function have been demonstrated in the field in: blue tits (*Cyanistes caeruleus*) [11], tree swallows (*Tachycineta bicolor*) [12], [13], house sparrows (*Passer domesticus*) [14], starlings (*Sturnus vulgaris*) [15] and collared flycatchers (*Ficedula albicola*) [16] although in the latter this was later shown to not be of genetic origin [17]. Body size has also been shown to be affected by nest-of-origin in several species [18]–[20]. Strong rearing environment effects on immune function in nestlings have been found in several species including; house sparrows [14], pied flycatchers (*F. hypoleuca*) [21], [22], collared flycatchers [16], great tits (*Parus major*) [23], [24], tree swallows [13] and starlings [15]. The brood size in which it is reared is one of the most obvious environmental effects for a young nestling, and an effect of rearing brood size on immune function has been demonstrated in both blue tits [11] and zebra finches (*Taeniopygia guttata*) [25]. These studies discriminate between the effects of genetic (+ early acting maternal effects) from environmental effects on nestling performance and in general have shown that both can play a role e.g. [20], [24].

Establishing the extent of genetic and/or environmental influences on offspring performance is valuable but insufficient to provide an answer to the discussion about whether direct or indirect benefits are gained through mate choice. If individuals are gaining direct benefits by choosing a high-quality mate, one would expect that adult ornaments should convey information about the immediate benefits to the offspring and that as a consequence offspring performance should be related to the ornamental traits of the social parents (i.e. the individuals rearing the offspring) [26]. In contrast, if individuals are gaining indirect benefits through their mate choice decisions one would expect to see relationships between the ornaments of the genetic parents and offspring performance.

There are few studies that simultaneously have disentangled the effects of rearing environment from genetic effects/natal environment, and tested for an effect of sexual selected ornaments on offspring fitness, Senar et al. demonstrated that the growth of blue tit chicks was related to the plumage yellowness of the social male and not to any measured features of the genetic parents, which suggests that females are gaining direct benefits through their choice of males with yellow plumage [26]. Similarly, in blue-footed boobies (*Sula nebouxii*) variance in chick condition was better predicted by the foot color of the social father than that of the genetic father [27]; this again suggests that direct benefits of mate choice are more significant than indirect benefits. While both direct and indirect effects have been shown to be important in mate choice in side-blotched lizards (*Uta stansburiana*) [28]. These studies are consistent with meta-analyses of (largely observational) data from several taxa, which have suggested that, in general, direct benefits are more substantial than indirect benefits of mate choice [29], [30].

Great tits are a common temperate, hole-nesting passerine, with both males and females displaying yellow carotenoid-based and black melanin-based colors in their plumage both of which appear to convey information about individual quality: males with large black breast stripes tend to be paired with early-breeding females [31] and this melanin-based display has been shown to be a signal of competitive ability [32]. Variation in the yellow, carotenoid-based coloration has been shown to correlate with aspects of health and condition in adult birds [33]–[36]. The expression of both the melanin-based breast stripe [37], [38] and the carotenoid display [36], [39] have been linked to parental quality. In addition to being used for coloration, carotenoid pigments are thought to have health benefits [40], [41], as they can act as free radical scavengers [42] and promote activation of the immune system [43]. There are several empirical studies demonstrating immunological benefits of dietary carotenoids in adult birds (mostly with captive populations and large doses of carotenoids e.g. [44], [45]; studies of free-living nestling great tits have failed to identify health benefits of supplemental carotenoids [46], [47].

In this study we performed experiments over four years to determine the influences of genetics (and early maternal effects) and environment on proxy measures of fitness in nestling great tits. Over three-years we conducted a cross-fostering field experiment to test for nest-of-origin and nest-of-rearing effects on nestlings immune function and body mass. To provide insight into the importance of direct versus indirect benefits we related these offspring performance measures to traits of the putative genetic parents (the adults at the nest-of-origin) and of the adults rearing the brood. We further manipulated the rearing environment through a provisioning experiment, providing food to adults in two years. In the final year, as carotenoids are the basis of the pigmentation in one of the ornamental traits in adults, we provided a direct carotenoid supplement to chicks. We would expect supplements to produce a positive effect on chick growth or immunocompetence.

## Materials and Methods

### Ethical Statement

This study was conducted under Home Office Licence 30/2244, both SRAP and MRE also had Home Office personal licences (numbers: 30/6759 and 30/3092) and MRE a BTO ringing permit (number: FA3499). The project was approved by University of Exeter’s ethics committee. Animal suffering was reduced as far as possible by minimising the number of procedures to which any individual was exposed, hence we decided not to take an additional blood sample to assess blood levels of carotenoids and we chose to assess color by removing feathers rather than exposing animals to the longer process that would have involved using the spectrometer directly on the bird. Work was conducted in Bagley Wood, which is owned by St John’s College, Oxford.

### General Methods

Data were collected in 1999 and from 2004–2006 in a temperate, mixed woodland, where great tits have been breeding in nest boxes for over 15 years; Bagley Wood, Oxfordshire UK (Grid Ref SP508024). We monitored all boxes regularly during the start of the season until eggs were found. Thereafter, boxes were checked daily to determine final clutch size and the start of incubation, to avoid disturbance during incubation once the number of eggs in the clutch had stopped increasing we estimated the expected day of hatching and then checked boxes daily during the nestling period. Chicks were ringed on day six using uniquely numbered British Trust for Ornithology rings. Nests were visited on day 13 to determine nestling mass (using digital scales (FS-125, My Weigh, GKI Technologies Phoenix AZ) accuracy 0.05 g) and the number of surviving offspring. Over the four years, a total of 393 breeding attempts and 2044 chicks were monitored (Table 1). Final samples sizes may differ from this and also may differ between analyses as not all birds were successfully measured for all traits and the sample size for any analysis is dictated by the set of birds for which all relevant measurements were taken.

**Table 1:** A summary of the experimental design, the traits assessed in each year and the annual sample sizes of nestlings and nests used in the experiment.

	1999	2004	2005	2006
Cross-fostering	X	X	X	
General food supplement		X	X	
Carotenoid supplement				X
Nestling immune function assay			X	X
Nestling body mass	X	X	X	X
Adult male breast stripe size & mass	X	X	X	X
Parental color traits & female mass		X	X	X
Number of nestlings	273	584	474	707
Number of nests	43	71	60	86

A shaded cell means that the design feature or measurement was utilised in that year.

### Experimental Design

A partial cross-fostering design was used in 1999, 2004 and 2005. On the day of hatching roughly half of the chicks in a nest were swapped with those from another nest that hatched on the same day. Nests to be matched were selected at random from those hatching on the same day, and brood sizes in the fostering nests remained the same. We distinguished fostered chicks from chicks remaining in their nest-of-origin by clipping a small amount of down from their backs. In total we had 279 chicks (43 broods) in our sample in 1999, 584 chicks from 137 broods in 2004, 474 chicks from 127 broods in 2005 and 707 chicks from 86 broods in 2006.

We provided two different food supplements. A general food supplement (Peanut Cake Tubes, C.J. Wildbird Foods Ltd., Shrewsbury UK) designed to supplement adult food intake was provided in 2004 and 2005, 66 broods received the supplement in 2004 while 42 broods were supplemented in 2005. At least one Peanut Cake Tube was hung close (<3 m) to half the nest boxes prior to the breeding season (provisioned nest boxes were determined at random each year) and maintained until the chicks had fledged. In 2006 nestlings were randomly assigned to one of two treatment groups (control or carotenoid-supplemented), this meant that there would be chicks within one nest in different treatment groups. Nestlings were fed the supplement during the period of most rapid growth on days 4, 5, 6, 8, 10, 11 and 12 following established techniques [48]. In brief, carotenoid supplements were prepared every 3 days in a 7.5% solution of leaf gelatin at 50°C in a 1 ml syringe and were quickly cooled in a refrigerator at 4°C and left to set. The resulting jelly mixture was administered by syringe into the nestlings’ mouths and was readily consumed by the birds. Syringes were bathed in ice inside thermos containers to keep them cool in the field before use. Carotenoid-supplemented chicks were fed 0.05 ml of 5 mg/ml OroGlo™ xanthophyll solution (Kemin, Des Moines, Iowa), containing 80% trans-lutein and 4% trans-zeaxanthin, containing 250 ug of xanthophylls, these are the commonest carotenoids in Lepidoptera larvae [49]. Assuming that nestlings only absorb approximately 20% of ingested carotenoids [50], this dose equates to around 50% of the naturally occurring mean daily carotenoid intake for a growing great tit chick [47]. This is much less than in has been used in previous carotenoid supplementation experiments on wild great tits [47], [51], [52] but is more likely to fall within the naturally occurring dietary range for nestling tits. Control nestlings received only the carrier gelatin mixture containing no carotenoids. In 2006, 309 chicks received a carotenoid supplement.

We were unable to assess the concentration of carotenoids in the blood, following supplementation. This would have necessitated taking additional blood samples from nestlings, which would have prejudiced the welfare of the chicks beyond what we felt was acceptable. However, in a parallel investigation on the same birds we found that carotenoid supplementation influenced the yellow ventral coloration of offspring (unpubl. data), which suggests that birds were absorbing the carotenoid supplement. In another study in which great tit chicks were fed carotenoids in a similar manner the chicks developed carotenoid-rich plumage [47].

### Nestling Immune Function

We assessed immunocompetence of nestlings in 2005 and 2006, using the PHA assay, which is a common and reliable method for assessing immune function in birds [24], [53], [54]. This technique induces an immune response (swelling) to a mitogen that is injected subcutaneously into the wing web, a swelling is produced at least partly as a result of infiltration of various immune factors from both the cell-mediated and acquired immune system [55]. The magnitude of this response is quantified as the thickness of the localised swelling around the injection site.

Thirteen-day old nestlings were injected with 0.01 mg of PHA-P (Sigma UK, L8754) dissolved in 0.02 ml phosphate-buffered saline solution (PBS) into the centre of the wing web (following Tschirren et al. [53]). Wing web swelling was measured to the nearest 0.01 mm using a pressure sensitive calliper (Teclock SM-112, Teclock, Japan) at 24 hours ±1 (n = 995) post injection. Readings were taken in triplicate and at 5 s intervals, to account for the initial rapid decrease in thickness as a result of the pressure of the calliper. Thickness was calculated as the mean of the three measurements. The difference between the thickness before and after the injection served as our measure of immunocompetence. Measurements were taken by SRAP. Our measurements of the wing web swelling had a high repeatability of 90%, (F _726,1454_ = 28.71, P<0.001) [56].

### Parental Traits

Adults were trapped in nest boxes using specifically designed spring traps when nestlings were at least 10 days old (overall we caught 156 males and 161 females). A small number of adults (ca. 10%) were found breeding in more than one year and these were included in the analysis only in the first year they were recorded. Adults were weighed to the nearest 0.05 g using digital scales (FS-125, My Weigh, GKI Technologies Phoenix AZ).

In 2004–2006, four to eight feathers were removed from standardised regions of the left and right carotenoid breast patches of adults. The feathers were overlaid to create a uniform patch from which we measured color with a reflectance spectrophotometer (ColortronTM; see Hill [57] for details). From the yellow curve obtained in the human-visible range, we obtained three color metrics – hue, saturation and brightness (HSB). Although vision in tit species is sensitive to ultra-violet wavelengths of light, we do not consider our method to be biased in quantifying carotenoid-derived color since xanthophyll pigments significantly affect light absorbance in human-visible wavelengths and not in the UV [26], [52], [58], [59]. HSB values were highly correlated within feather patches and individuals (all P<0.001 for males and females with r^2^ ranging from 0.25 to 0.73), so we used Principal Component Analysis (PCA) to derive un-correlated color components, which we refer to as “yellowness” (PC1) as it was almost equally loaded on saturation and hue and “yellow brightness” (PC2) as it was heavily loaded on brightness (see Table S1). PC1 explained 63.4% of the variance in the HSB measurements of yellow plumage in females and 66.4% in males, while PC2 explained a further 27.7% of the variance in females and 23.2% in males. PCA was conducted using the princomp procedure in R [60].

For the melanin breast stripe patch, saturation and brightness (not hue, which is irrelevant for black or white shades) were calculated in the manner described above and since color components were again strongly correlated (r^2^ 0.47–0.60, P<0.001), we combined them into one PC which we will refer to as “stripe blackness” and was equally loaded on both brightness and saturation and explained 79.7% of the variance in the colour of the breast stripe of females and 71% in males (Table S1). The correlation matrix showing the strengths of the relationships between male colour variables and female colour variables are shown in table 2. Breast stripe size was measured for males and calculated by taking the sum of the widths at five standardised points following [9].

**Table 2:** The colour variables from the same individual are uncorrelated.

Males	Yellow brightness	Yellowness	Stripe blackness
Yellow brightness	–		
Yellowness	1.24×10^−16^ (P>0.99)	–	
Stripe blackness	0.0013 (P = 0.99)	0.0018 (P = 0.99)	–
Stripe width	−0.064 (P = 0.54)	−0.054 (P = 0.60)	0.16 (P = 0.12)
Females	Yellow brightness	Yellowness	Stripe blackness
Yellow brightness	–		
Yellowness	4.96×10^−17^ (P>0.99)	–	
Stripe blackness	0.11 (P = 0.26)	−0.18 (P = 0.07)	–
Mass	0.0058 (P = 0.95)	0.115 (P = 0.24)	0.024 (P = 0.81)

Table shows Pearson’s correlation coefficients calculated between the traits of male and female great tits. None of these relationships differ significantly from chance, although the relationship between female yellowness and stripe blackness is close to significance. Note that as yellowness and yellow brightness are PC1 and PC2 of the yellow plumage one would expect them to be uncorrelated. Samples sizes for these relationships are 94 males and 105 females.

A summary of the experimental design employed, which traits were measured in each years and sample sizes of nestlings and nests is shown in table 2. Unfortunately, in this study we were unable to assess parentage directly using genetic techniques. It is known that extra-pair paternity occurs in great tits, estimated in a nearby population to result in 13–14% of offspring being unrelated to their social father [61]–[63]. It is therefore possible that any failure to detect relationships between offspring performance and the traits of nest-of-origin males could be due the level of extra-pair paternity. Relationships between offspring performance and traits of putative mothers (no examples of offspring being unrelated to their putative mother were reported by either Blakey [62] or Patrick et al. [63]) and the traits of rearing adults will be unaffected by this possible source of error.

### Data Analysis

Our study included both experimental components (manipulation of the environment through adult food supplementation and directly supplementing chicks with carotenoids and cross-fostering chicks) and observational components where we looked to explain variance in chick performance by reference to variance in characteristics of their social or genetic parents. The presence of a significant element of hypothesis testing and our desire to adopt a simple, unified analytical process led us to adopt an approach in which we fitted mixed-model ANOVA followed by model simplification to yield a minimal adequate model [64].

Data were analysed using mixed model ANOVA implemented using the glmer procedure in the lme4 library for R [60], [65]. Models used chick body mass and the size of the wing web swelling in response to PHA injection as dependent variables. Nests were grouped into cross-foster groups, normally composed of two nests between which nestlings were exchanged. Following Brinkhof et al. [24] we used a mixed model analysis with data included for each nestling. The first set of models used a random model only, with random effects of: nest-of-origin; the nest in which the chick was raised and the cross-foster group (nest-of-origin and rearing nest were both nested within cross-foster group). The second set of models included, in addition to the same random model, a fixed model that included as fixed independent effects; the adult traits (as described above) for the male and female at the nest-of-origin and the male and female at the rearing nest; the mass of the chick and the brood size in which it was raised; a binomial code to identify whether or not the nest in which the chick was raised was provided with supplemental food (provisioned); and a binomial code identifying whether or not the chick had been fed a carotenoid supplement. As color measurements were only taken in 2004–2006, two models had to be run for each dependent variable. One included data for all years and included data on variables for which data were collected in all years (male breast stripe size, brood size, nestling mass). The other included data from years 2004–2006 and included a wider range of data on parental variables (adding female body mass, male and female stripe blackness, yellowness and yellow brightness). Therefore four models were run on each dependent variable. Model simplification involved hierarchical step-wise deletion, with those terms explaining the least variance (least significant in the model) in the response being removed first. The final model was accepted when all remaining fixed-effect terms explained significant variance in the dependent variable. Model residuals were checked for heteroscedasticity and that they conformed to a normal distribution. The significant (P<0.05) factors in the fixed model from the full (unsimplified) model are reported in Appendix S1 and those derived from the maximally simplified model are reported in the text.

There is debate about the appropriate denominator degrees of freedom in complex mixed model ANOVAs, see for example (https://stat.ethz.ch/pipermail/r-help/2006-May/094765.html). In this paper we have reported significance tests for the terms in our models. For the fixed model we have used a denominator degrees of freedom obtained by subtracting appropriate degrees of freedom for all the fixed and random effects in the model (i.e. one for continuous variables and (number of levels –1) for categorical variables). For the random model we used Satterthwaite’s approximation [64], [66], [67] to determine appropriate degrees of freedom.

## Results

### Immunocompetence

Immunocompetence in nestlings was measured in two years, in both of which we have data on adult morphological traits (Table 1). Data are available for this analysis from 1,181 nestlings in 146 broods.

**Figure 1:**
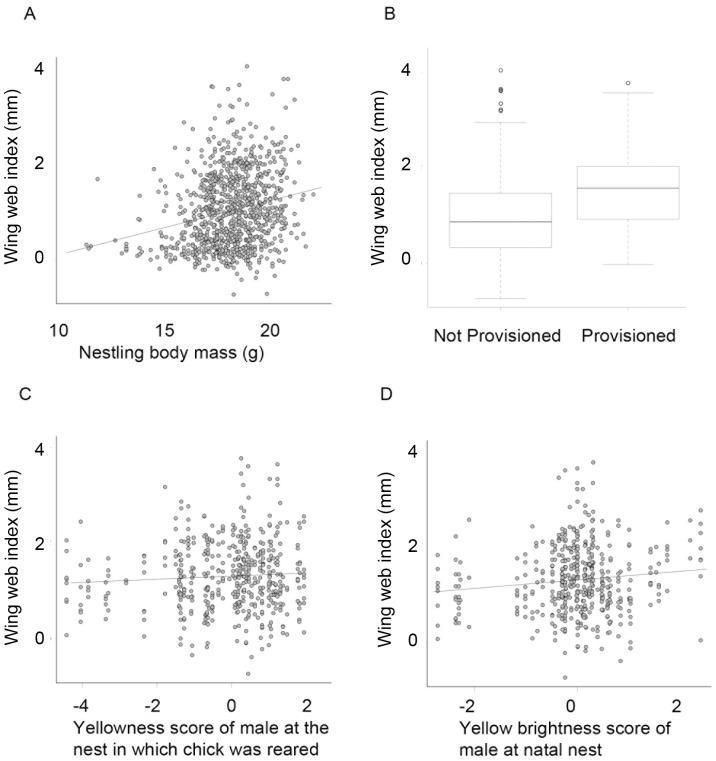
Thickness of the wing web swelling 24 hours after injection with PHA varied with: A) nestling body mass; B) adult food supplementation (boxplot giving median and interquartile range); C) plumage yellowness of the rearing father; and, D) yellow plumage brightness of the nest-of-origin male. Raw data are presented.

**Figure 2:**
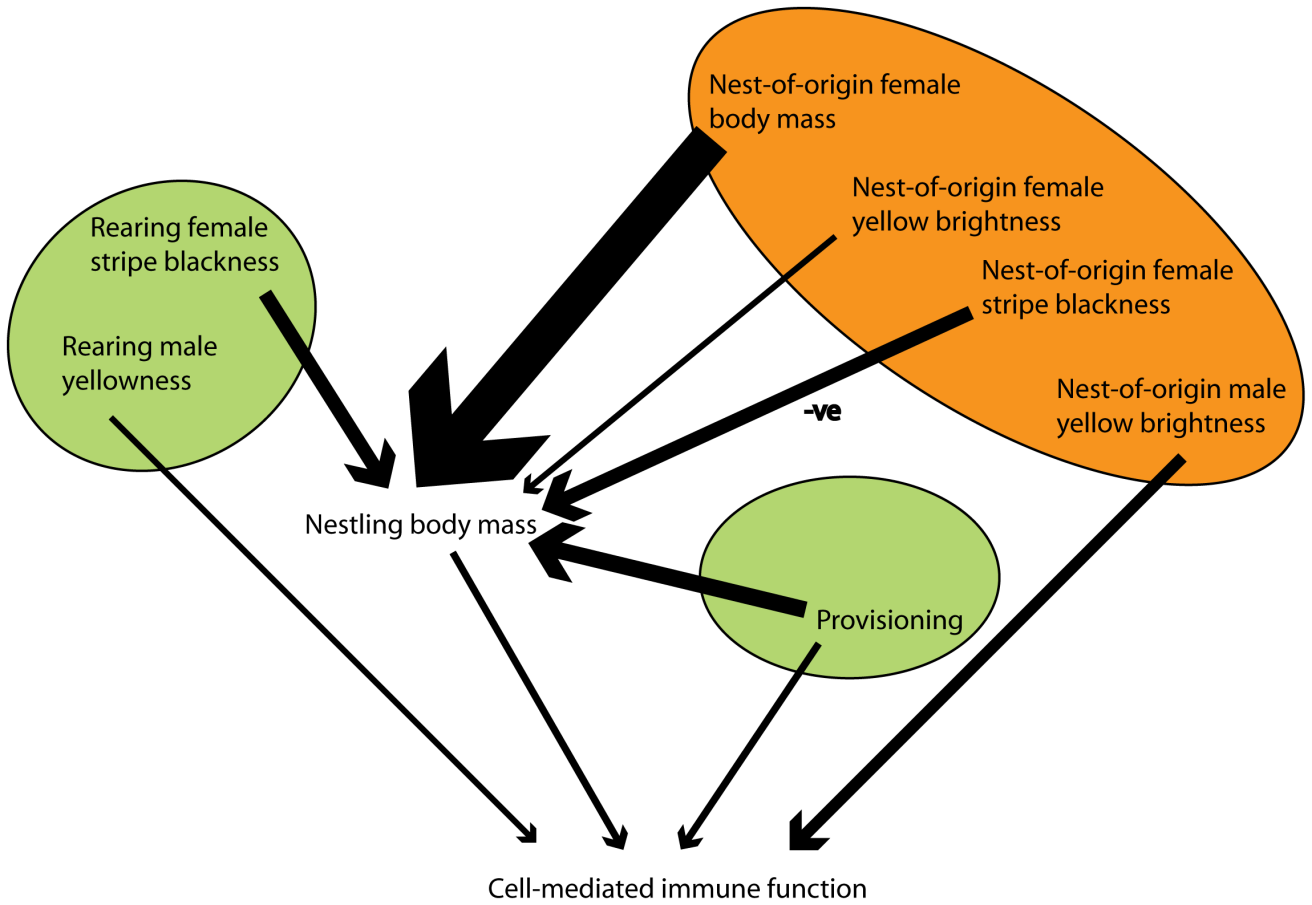
Summary diagram showing the effects of the significant independent factors on nestling immunocompetence and body mass in this study. Factors associated with the rearing environment are shaded green, those with the natal environment are in orange. Arrow width reflects the strength of the association (F value) between the two variables.

After model simplification, supplemental provisioning (F_1,192_ = 4.47, P = 0.035), nestling body mass (F_1,192_ = 5.19, P = 0.024), the yellow plumage brightness of the nest-of-origin male (F_1,192_ = 9.93, P = 0.002) and the plumage yellowness of the rearing male (F_1,192_ = 5.25, P = 0.023) explained significant variance in nestling immunocompetence, with yellower males raising nestlings that have greater PHA-induced swelling than less-yellow males, and males with brighter yellow plumage being the putative fathers of nestlings with higher immunocompetence (Figure 1, Figure 2). The variance components of both rearing nest and nest-of-origin were small (4.28×10^−9^ and 2.14×10^−9^ respectively) but the variance component of the cross-foster group was large (0.137) and explained a substantial amount of the total variance (Table S2).

Carotenoid supplementation did not explain significant variance in immune function in either a full model (F_1,77_ = 1.38, P = 0.25) or a simplified model (i.e. it dropped out of the model during the simplification process). We found little evidence of year-to-year repeatability of performance in nest boxes. This suggests that the environment quality around each nestbox changes significantly between years (Appendix S2).

### Day 13 Body Mass

Nestling body mass was measured in all four years, and data are available from 2,038 nestlings in 260 broods. A full set of parental morphological traits was taken in three years. Therefore, we have conducted two analyses, one including data from all four years and only using parental traits measured in all four years and one that only uses data from three years but includes the full set of parental morphological traits.

**Figure 3:**
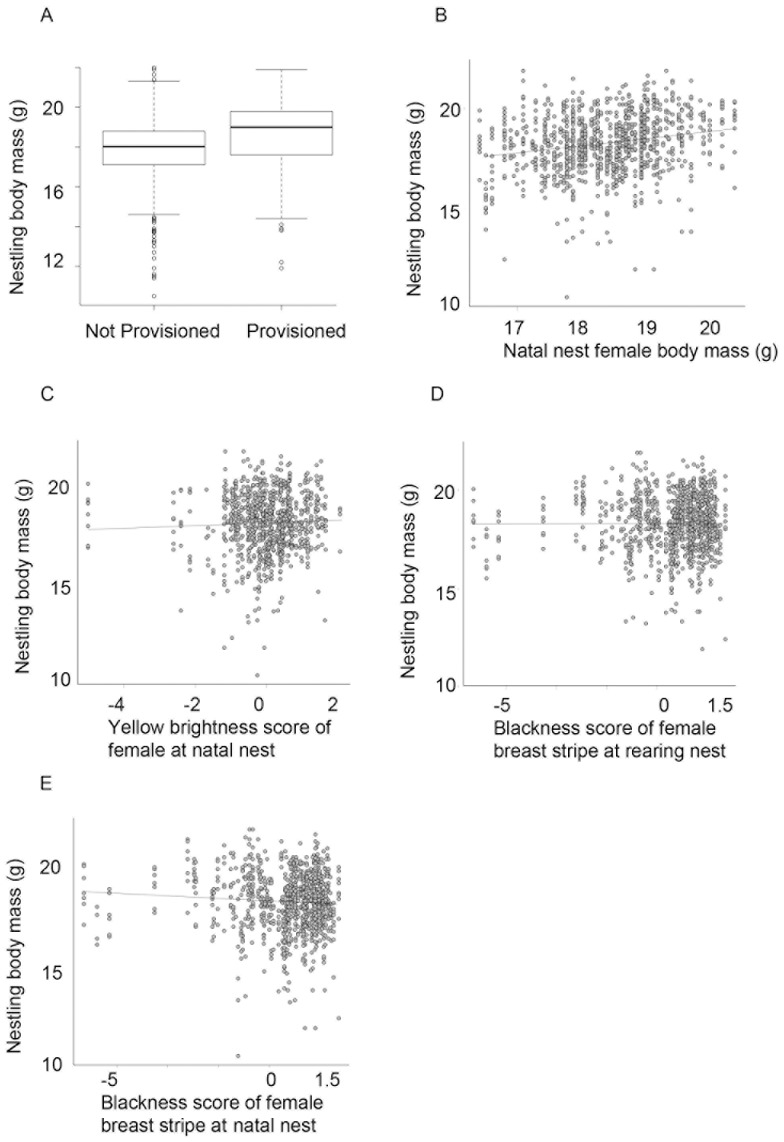
Nestling body mass was influenced by: A) food supplementation of attending parents (boxplot giving median and interquartile ranges); B) body mass of the nest-of-origin mother; C) yellow plumage brightness of the nest-of-origin mother; D) breast-stripe blackness of the rearing female; E) breast-stripe blackness of the nest-of-origin mother. Raw data are presented.

**Figure 4:**
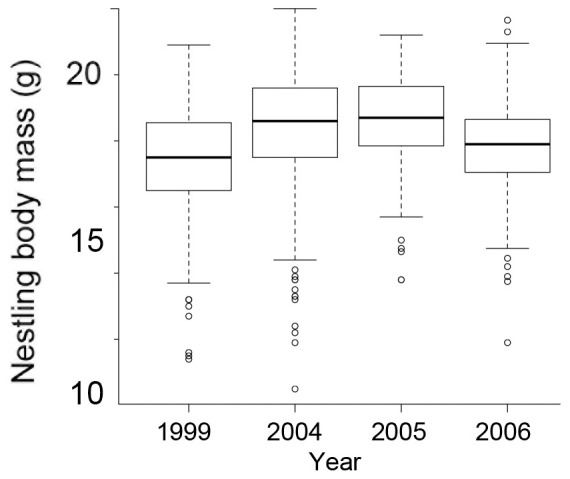
Effect of year on body mass, indicating that nestlings were lighter in 1999 and 2006 than in 2004 and 2005 (boxplot gives median and interquartile range).

After model simplification of the model associated with the four-year dataset, adult food supplementation was the only independent variable explaining significant variance in nestling body mass (F_1,1084_ = 14.20, P<0.001), such that nestling body mass was positively affected by increased food provisioning (Figure 3). Examination of the cross-foster group coefficients suggests that there was a year effect on body mass, with chicks being lightest in 1999, followed by 2006, while nestlings were heaviest in 2004/5 (Figure 4). The variance components of both the rearing nest and the nest-of-origin (both nested within cross-foster group) were small (1.83×10^−7^ and 8.34×10^−8^ respectively), although the variance component associated with the cross-foster group factor was large (2.06) (Table S2).

After model simplification of the three-year dataset, adult food supplementation (F_1,654_ = 13.98, P<0.001), the nest-of-origin female stripe blackness (F_1,654_ = 13.47, P<0.001), body mass of the nest-of-origin female (F_1,654_ = 35.66, P<0.001), yellow plumage brightness of the nest-of-origin female (F_1,654_ = 4.78, P = 0.029), and stripe blackness of the rearing female (F_1,654_ = 12.38, P<0.001) explained significant variance in nestling body mass. Specifically, we found that nestlings were heavier when the nest was experimentally provisioned, when the nest-of-origin female was heavy and had brighter yellow plumage and when they were reared by a female with a blacker breast stripe. In contrast nest-of-origin females with blacker breast stripes produced eggs that developed into lighter chicks (Figure 3). The relative sizes of the variance components associated with the nest-of-origin and rearing nest was again very small (2.64×10^−7^ and 1.34×10^−9^ respectively) compared to that of the cross-foster group (1.063). Cross-foster group consistently explained a substantial amount of the variance in the random model.

Carotenoid supplementation did not explain significant variance in nestling body mass in either a full model (F_1,270_ = 0.33, P = 0.56) or a simplified model (i.e. it dropped out of the model during the simplification process for either the three or four year datasets. We found little evidence of year-to-year repeatability of performance in nest boxes. This suggests that the environment around each nestbox changes significantly between years (Appendix S2).

## Discussion

Females gain through mate choice either immediately via direct benefits (e.g. enhanced parental care, territory quality) or through indirect benefits (e.g. enhanced viability of their offspring; [4]). Both theory [68], [69] and data [29], [30] suggest that direct benefits are usually large in comparison to indirect benefits. This is supported by our analysis, the cross-foster group random effect was associated with a significant variance component in all analyses. The only factors that would be shared by the nests within a cross-foster group are associated with time in the season and year. There should be no genetic or spatial association between nests within a cross-foster group, which was assigned solely on the basis of the time at which the chicks hatched. Our analysis of the fixed effects suggests that female great tits could be gaining both direct and indirect fitness benefits on offspring immunocompetence by choosing males based on aspects of the yellow ventral coloration, as there are similarly sized significant effects of the yellow plumage both of males at the nest-of-origin and of rearing males (Figure 2). Direct effects are plausible because carotenoid-based pigmentary colors often reflect condition [34], [35], [70] and, at least in the closely related blue tit, reflect ingestion of carotenoid-rich caterpillar prey [26]. In great tits the health and condition of the offspring has been shown to be correlated with the yellowness of the ventral plumage of the male providing parental care [33], and an experimental study in blue tits showed that chick growth rates were better predicted by the yellowness of the foster father than by traits of the genetic parents [26]. In parallel observations of the plumage color of the chicks in this study we found that carotenoid supplemented chicks developed yellower plumage (unpublished data). There is also evidence in sticklebacks (*Gasterosteus aculeatus*) that dietary carotenoids enhance parental care [71]. The rearing-male effect suggests that there is a link, possibly through the environment around the rearing nest and/or male parental care, between male plumage color and offspring immunocompetence. However, the nest-of-origin male effect also suggests that females mating with males whose yellow plumage is brighter may gain indirect fitness benefits through enhanced immunocompetence of nestlings. Therefore, the brightness of the male’s plumage might signal some heritable component of immunocompetence and/or that there are maternal effects that are biased with respect to male plumage and which influence immunocompetence in offspring. It is worth noting that none of the traits measured in females predicted significant variance in offspring immune function.

Especially in species with significant maternal investment females can strongly influence offspring development. There is an increasing realisation that non-genetic, maternal effects can play a significant role in offspring development [72] and in birds this may occur through female investment in eggs [73]. In the present study, several traits of the nest-of-origin (genetic) mother significantly predicted nestling body mass, although none were significant predictors of nestling immunocompetence. Heavier females were associated with heavier chicks near the end of the nestling stage, as were females with brighter yellow plumage. It is perhaps unsurprising that heavy females produce heavy chicks – these females might lay larger eggs [74], [75], which could give rise to larger chicks and/or they may pass on genes for greater body size [76]. The brightness of a female’s yellow plumage may reflect her ability to nutritionally or physiologically accumulate carotenoids and perhaps allocate them to egg yolk, thereby aiding offspring development and growth [77]. It is worth noting that, as nestling mass was correlated with their immunocompetence, logically any variable that significantly influences body mass would be likely to be related to immunocompetence (Figure 2). No traits measured in males predicted significant variance in nestling body mass. These results imply that males might benefit from selecting large females with bright yellow plumage as mates, this is as yet unrecorded in great tits although it is known that attractive males tend to be mated to early breeding females [31]. If males were to select large, bright yellow plumaged females one would predict that they may gain an advantage in producing larger chicks, which will tend to survive well to the next breeding season [78]. Research into male choice of female traits is relatively undeveloped but is likely to be more widespread than the literature suggests [79].

Food supplementation has been used frequently as an experimental tool in avian ecology. It has been shown that food supplements often result in breeding starting earlier in the season [80] and often increases breeding success [80], [81]; although unexpected negative effects can also occur, for example [82] found that clutch and brood size declined in continuously supplemented areas. There have been many examinations of the effect of food supplementation on nestling growth, with almost all (as in this study) showing a positive effect on growth [80]. We are unaware of any test of the effect of supplementation on nestling immunocompetence. When we manipulated the rearing environment by placing commercially available adult food close to nestboxes, we significantly increased both nestling growth and immunocompetence. We never witnessed adults taking the supplement into the nestbox and so they probably did not feed it to the chicks, although the adults were frequently seen to utilise the artificial food to feed themselves (pers obs), this is unsurprising as the peanut food would be unsuitable as a nestling diet. The fact that chicks raised by provisioned parents had both higher body mass and immunocompetence than chicks raised by unprovisioned parents suggests that the supplemental food supply allowed provisioned adults to spend more time feeding their young than unprovisioned adults. It is worth noting that our analysis of immunocompetence also included the effect of chick mass and so the effect of supplements on immunocompetence was additional to its effect on nestling body mass. This suggests that either the rearing birds are able to feed their brood food that enhances immunocompetence without affecting mass or they are able to find food items that enable their offspring specifically to boost their immunocompetence. The effect of food supplementation on immunocompetence may provide an explanation for the reported effects on nestling survival [80].

Like the two previous studies of free-living nestling great tits, we found no significant impact of supplemental carotenoids on immunocompetence or body mass [46], [47]. It is possible that, in animals (like tit nestlings) that naturally consume high concentrations of carotenoids (in this case from their caterpillar prey; [47]), circulating carotenoid levels are sufficient in all animals to meet or exceed those required for proper antioxidant defence and immune system functioning. Perhaps in this situation it is not possible to manipulate the system by providing a carotenoid supplement.

In our study the cross-foster group term consistently explained a high amount of the variance in all the models. As nests within a cross-foster group always contained nestlings that hatched on the same day, foster-group effects could be due to annual and/or seasonal temporal variability in environmental conditions, but not spatial effects since nests within a cross-foster group could be located anywhere within the forest. The influence of year is readily apparent, with strong effects on both nestling immunocompetence and body mass, while seasonal effects on great tit breeding success are well known [83]. Our results suggest that neither nest-of-origin nor rearing nest explained much additional variance over and above the cross-foster group factor. This differs somewhat from [24] who suggested that there were significant effects of both factors in addition to cross-foster group. This may be partly due to the fact that, by assessing the effects of the traits of the putative parents and the rearing adults, we accounted for the variance explained by these factors on nestling performance measures. This cannot be the entire explanation, however, as similar differences between the two studies can be seen when there is no fixed model. One difference between the two studies is the time scale over which data were collected; ours was conducted over four years (although immunocompetence was only assessed in two) while Brinkhof et al.’s lasted one year [24]. This would mean that the cross-foster group factor in our study included annual as well as seasonal variation, increasing the variance in the cross-foster group factor above that in Brinkhof et al that would have only reflected seasonal effects [24]. This may be supported by the fact that we found little consistency in the performance of offspring raised in the same box between years, which suggests that the environment quality around the nestbox changes between years, or the individuals breeding in the nestbox differ significantly in their ability to take advantage of the environment. The swapping of chicks between nests occurred on the day on which the chicks hatched. There was therefore limited opportunity (maximum ca. 12 hours) for the nest-of-origin parents to feed the chicks before they were fostered into the rearing nest. In theory, we could have been prevented this by swapping eggs rather than chicks, but the chicks would have been indistinguishable after they had hatched. In addition in our experiment any pre-hatching maternal effects would remain confounded with genetic effects in our experiment, maternal effects through investment in eggs does occur in birds [73], [84]–[86]. If this was important in great tits it might be possible that the nest-of-origin effects described here (or in a study that swapped eggs) could be due to such early acting maternal effects. However, it is almost impossible to conceive how this might be overcome given that swapping embryonic young before the deposition of the egg seems extremely difficult.

In conclusion, we found that manipulations of the rearing environment in the form of food supplements for adults had significant and consistently positive effects on offspring performance. There was a significant effect of the ventral coloration of both foster and nest-of-origin males on nestling immune function, which is consistent with the interpretation that females could gain both direct and indirect benefits by mating with males signalling in different ways. Several traits of the female at the nest-of-origin – body mass, the brightness of her yellow plumage and the blackness of her breast stripe – explained significant variance in chick body mass, as did the breast-strip blackness of the rearing female. These effects are consistent with the coloration of females predicting differential investment in eggs and/or post-hatch rearing of young [87].

## Supporting Information

Table S1 **Results of the principal component analysis of plumage color traits in this study.** Hue, saturation and brightness (HSB) scores were derived from the single yellow curve in the human-visible range from patches of feathers taken from standardised patches of the right and left sides of the bird’s yellow breast patch. Black color was assessed in a similar way by measuring saturation and brightness from one patch of feathers taken from the bird’s black breast stripe. Principal components analysis has been used to reduce the correlated HSB scores down to uncorrelated principal components.
(DOCX)

Table S2 **Results of nested analyses of variance that tested effects of rearing environment and nest-of-origin on chick immunocompetence and body mass, utilising data from all nestlings.** a) Random effects model. b) Mixed model, including both random and fixed effects; fixed effects were divided into those that related to the chick’s rearing environment (i.e. traits of the rearing parents, supplemental provisioning) and to its nest of origin (i.e. traits of the nest of origin parents) and for the analysis of immunocompetence to the individual chick’s body mass. Nest of rearing and origin were nested within foster group. * Chick immunity was not measured in 2004, so these analyses use data from one fewer year than the analyses on chick mass.
(DOCX)

Appendix S1 **The full (unsimplified) ANOVA models.**
(DOCX)

Appendix S2 **Repeatability of nest site effects.**
(DOCX)
