# Supplementary material for: Environmental and Parental Influences on Offspring Health and Growth in Great Tits (Parus major)
Source: PLoS One. 2013 Jul 30;8(7):e69695. doi: 10.1371/journal.pone.0069695 (PMC3728352; doi:10.1371/journal.pone.0069695)
Supplement: Table S1 — Results of the principal component analysis of plumage color traits in this study. Hue, saturation and brightness (HSB) scores were derived from the single yellow curve in the human-visible range from patches of feathers taken from standardised patches of the right and left sides of the bird’s yellow breast patch. Black color was assessed in a similar way by measuring saturation and brightness from one patch of feathers taken from the bird’s black breast stripe. Principal components analysis has been used to reduce the correlated HSB scores down to uncorrelated principal components. (DOCX) [file pone.0069695.s001.docx]

Table S1. Results of the principal component analysis of plumage color traits in this study. Hue, saturation and brightness (HSB) scores were derived from the single yellow curve in the human-visible range from patches of feathers taken from standardised patches of the right and left sides of the bird’s yellow breast patch. Black color was assessed in a similar way by measuring saturation and brightness from one patch of feathers taken from the bird’s black breast stripe. Principal components analysis has been used to reduce the correlated HSB scores down to uncorrelated principal components.

a) Variance explained by each principal component.

| Yellow feathers | Females | | | Males | | |
| --- | --- | --- | --- | --- | --- | --- |
|  | Eigenvalues | | | Eigenvalues | | |
| Component | Total | % variance | Cumulative % | Total | % variance | Cumulative % |
| 1 | 1.37 | 63.4 | 63.4 | 1.41 | 66.4 | 66.4 |
| 2 | 0.91 | 27.7 | 91.1 | 0.83 | 23.2 | 89.6 |
| 3 | 0.52 | 8.9 | 100 | 0.56 | 10.4 | 100 |
| Black stripe | Females | | | Males | | |
|  | Eigenvalues | | | Eigenvalues | | |
| Component | Total | % variance | Cumulative % | Total | % variance | Cumulative % |
| 1 | 1.26 | 79.7 | 79.7 | 1.19 | 71.0 | 71.0 |
| 2 | 0.64 | 20.2 | 100 | 0.76 | 29.0 | 29.0 |

b) Loadings of each color trait on the principal components used in the analysis; PC1 (predominantly saturation and hue) and PC2 (mainly brightness) were used for yellow colors, while PC1 (a combination of brightness and saturation) was used for the black stripe.

| Yellow feathers | Females | | | Males | |  |
| --- | --- | --- | --- | --- | --- | --- |
| Component | PC1 | PC2 | | PC1 | PC2 |  |
| Brightness | 0.40 | 0.91 | | 0.50 | 0.84 |  |
| Saturation | 0.64 | -0.34 | | 0.63 | -0.19 |  |
| Hue | 0.66 | -0.23 | | 0.59 | -0.51 |  |
| Black stripe | Females | | Males | | | |
| Component | PC1 | | PC1 | | | |
| Brightness | 0.71 | | 0.71 | | | |
| Saturation | 0.71 | | 0.71 | | | |
