# Supplementary material for: Environmental and Parental Influences on Offspring Health and Growth in Great Tits (Parus major)
Source: PLoS One. 2013 Jul 30;8(7):e69695. doi: 10.1371/journal.pone.0069695 (PMC3728352; doi:10.1371/journal.pone.0069695)
Supplement: Table S2 — Results of nested analyses of variance that tested effects of rearing environment and nest-of-origin on chick immunocompetence and body mass, utilising data from all nestlings. a) Random effects model. b) Mixed model, including both random and fixed effects; fixed effects were divided into those that related to the chick’s rearing environment (i.e. traits of the rearing parents, supplemental provisioning) and to its nest of origin (i.e. traits of the nest of origin parents) and for the analysis of immunocompetence to the individual chick’s body mass. Nest of rearing and origin were nested within foster group. * Chick immunity was not measured in 2004, so these analyses use data from one fewer year than the analyses on chick mass. (DOCX) [file pone.0069695.s002.docx]

Table S2. Results of nested analyses of variance that tested effects of rearing environment and nest-of-origin on chick immunocompetence and body mass, utilising data from all nestlings. a) Random effects model. b) Mixed model, including both random and fixed effects; fixed effects were divided into those that related to the chick’s rearing environment (i.e. traits of the rearing parents, supplemental provisioning) and to its nest of origin (i.e. traits of the nest of origin parents) and for the analysis of immunocompetence to the individual chick’s body mass. Nest of rearing and origin were nested within foster group. * Chick immunity was not measured in 2004, so these analyses use data from one fewer year than the analyses on chick mass.

a) No fixed model

| Dataset | Dependent variable | Source | F | p | Degrees of freedom | Variance Component |
| --- | --- | --- | --- | --- | --- | --- |
| 4 year dataset | Chick mass | Cross-foster group | 17.76 | < 0.001 | 124 | 2.24 |
|  |  | Rearing nest | 0.99 | 0.488 | 135 | 2.14x10^-7^ |
|  |  | Nest of origin | 0.99 | 0.487 | 128 | 8.69x10^-8^ |
|  |  | Error |  |  | 1085 | 1.55 |
| 3 year dataset | Chick immunity* | Cross-foster group | 5.13 | < 0.001 | 52 | 0.182 |
|  |  | Rearing nest | 1 | 0.491 | 99 | 6.00x10^-9^ |
|  |  | Nest of origin | 1 | 0.493 | 108 | 5.31x10^-9^ |
|  |  | Error |  |  | 232 | 0.409 |
|  | Chick mass | Cross-foster group | 13.98 | < 0.001 | 53 | 1.91 |
|  |  | Rearing nest | 1 | 1 | 99 | 3.67x10^-7^ |
|  |  | Nest of origin | 1 | 1 | 108 | 5.77x10^-8^ |
|  |  | Error |  |  | 660 | 1.42 |

b) With fixed model

| Dataset | Dependent variable | Source | F | p | Degrees of freedom | Variance Component |
| --- | --- | --- | --- | --- | --- | --- |
| 4 years with data on limited set of parental traits | Chick mass | Cross-foster group | 14.71 | < 0.001 | 124 | 2.06 |
|  |  | Rearing nest | 1 | 0.487 | 135 | 1.83x10^-7^ |
|  |  | Nest of origin | 1 | 0.486 | 128 | 8.34x10^-8^ |
|  |  | Fixed effects |  |  | 1 |  |
|  |  | Rearing nest effects | 14.2 | < 0.001 | 1 |  |
|  |  | Nest of origin effects | - | - | - |  |
|  |  | Error |  |  | 1084 | 1.55 |
| 3 years with data on larger set of parental traits | Chick immunity | Cross-foster group | 4.15 | < 0.001 | 53 | 0.137 |
|  |  | Rearing nest | 1 | 0.491 | 99 | 4.28x10^-9^ |
|  |  | Nest of origin | 1 | 0.493 | 108 | 2.14x10^-9^ |
|  |  | Fixed effects |  |  | 4 |  |
|  |  | Rearing nest effects | 9.71 | < 0.001 | 2 |  |
|  |  | Nest of origin effects | 9.93 | 0.002 | 1 |  |
|  |  | Chick body mass | 5.19 | 0.024 | 1 |  |
|  |  | Error |  |  | 192 | 0.403 |
|  | Chick mass | Cross-foster group | 8.83 | < 0.001 | 92 | 1.063 |
|  |  | Rearing nest | 1.00 | 0.486 | 98 | 2.64x10^-7^ |
|  |  | Nest of origin | 1.00 | 0.487 | 114 | 1.34x10^-9^ |
|  |  | Fixed effects | 80.26 | < 0.001 | 5 |  |
|  |  | Rearing nest effects | 26.36 | < 0.001 | 2 |  |
|  |  | Nest of origin effects | 53.90 | < 0.001 | 3 |  |
|  |  | Error |  |  | 654 | 1.31 |
