# Supplementary material for: Environmental and Parental Influences on Offspring Health and Growth in Great Tits (Parus major)
Source: PLoS One. 2013 Jul 30;8(7):e69695. doi: 10.1371/journal.pone.0069695 (PMC3728352; doi:10.1371/journal.pone.0069695)
Supplement: Appendix S1 — The full (unsimplified) ANOVA models. (DOCX) [file pone.0069695.s003.docx]

Appendix S1. The full (unsimplified) ANOVA models.

Immunocompetence: In the full model the only independent variables that explained significant variance in immunocompetence were supplemental provisioning (F_1,77_ = 6.18, P = 0.015), nestling body mass (F_1,77_ = 5.89, P = 0.017), and the mass of the nest of origin female (F_1,77_ = 4.28, P = 0.042).

Body mass four year model: Considering the full model we found that the brood size in which the nestling was reared (F_1,626_ = 9.73, P = 0.002) adult food supplementation (F_1,626_ = 9.76, P = 0.002) and breast stripe size of the rearing male (F_1,626_ = 4.32, P = 0.038) explained significant variance in nestling body mass.

Body mass three year model: When we considered the full model, we found effects of brood size (F_1,270_ = 5.67, P = 0.018), adult food supplementation (F_1,270_ = 17.12, P < 0.001), yellow plumage brightness of the nest-of-origin female (F_1,270_ = 7.75, P = 0.006), yellow plumage brightness of the rearing female (F_1,270_ = 5.98, P = 0.015), plumage yellowness of the rearing female (F_1,270_ = 6.32, P = 0.013) stripe blackness of the rearing female (F_1,270_ = 17.58, P < 0.001), plumage yellowness of the rearing male (F_1,270_ = 5.51, P = 0.019) and the body mass of the nest-of-origin female (F_1,270_ = 7.95, P = 0.005).
