# Supplementary material for: Environmental and Parental Influences on Offspring Health and Growth in Great Tits (Parus major)
Source: PLoS One. 2013 Jul 30;8(7):e69695. doi: 10.1371/journal.pone.0069695 (PMC3728352; doi:10.1371/journal.pone.0069695)
Supplement: Appendix S2 — Repeatability of nest site effects. (DOCX) [file pone.0069695.s004.docx]

Appendix S2. Repeatability of nest site effects.

Nestling immune function. The fitted values for each nest of this analysis were taken and tested to determine whether or not nestling immunocompetence was consistent within nestboxes across different years. There was no significant relationship between the mean immunocompetence of chicks that originated from a nest box in one year and those that originated from the same box in other years (F_7,8_ = 0.10, P=0.99), and the repeatability was low ([Lessells and Boag 1987](#_ENREF_1)). Similarly there was no significant effect of the nestbox in which nestlings were reared in different years on immunocompetence (F_10,11_ = 0.34, P=0.95), and again the repeatability was low (-0.49).

Day 13 body mass. The fitted values for each nest of this analysis were taken and tested to determine whether a relationship existed between the body masses of nestlings originating from the same box in different years. There was no significant relationship between the body mass of nestlings originating from a nest box in one year and those originating from the same box in other years (F_20,25_ = 1.20, P=0.32). The repeatability of nestling body mass for nestlings originating from the same nest in different years was low (0.06) ([Lessells and Boag 1987](#_ENREF_1)). Similarly there was no significant effect of rearing nest on the body mass of chicks reared in the same nest box in different years (F_24,29_ = 1.18, P=0.33), again with a low repeatability (0.06).

Lessells, C. M. and P. T. Boag. 1987. Unrepeatable repeatabilities: a common mistake. Auk **104**:116-121.
